# P-1003. Evolutionary Differences within the Epidemic Clostridioides difficile Strain Identified as PCR-Ribotype 027

**DOI:** 10.1093/ofid/ofaf695.1200

**Published:** 2026-01-11

**Authors:** Andrew M Skinner, Laurica A Petrella, Jennifer Cadnum, Adam K Cheknis, Munok Hwang, Hosoon Choi, Charlesnika T Evans, Chetan Jinadatha, Larry K Kociolek, Curtis Donskey, Dale N Gerding, Matthew H Samore, Stuart Johnson

**Affiliations:** University of Utah, Salt Lake City, UT; Edward Hines Jr VA Hospital, Hines, Illinois; Northeast Ohio VA Medical Center, Cleveland, Ohio; Edward Hines Jr. VA Hospital, Hines, Illinois; Central Texas Veterans Health Care System, Temple, Texas; Central Texas Veterans Health Care System, Temple, Texas; Northwestern University and VA, Hines, Illinois; Central Texas Veterans Healthcare System, Temple, Texas; Ann & Robert H. Lurie Children's Hospital of Chicago, Chicago, IL; Cleveland VA Hospital, Cleveland, Ohio; Edward Hines, Jr. Veterans Affairs Hospital, Hines, Illinois; University of Utah, Salt Lake City, UT; Hines VA Hospital and Loyola University Medical Center, Hines, Illinois

## Abstract

**Background:**

In the early 2000s, the epidemic strain identified as PCR Ribotype 027 (RT027) and multilocus sequence type (MLST) 1 was the dominant strain group accounting for >70% of CDI in some institutions. Notably, the strain group was fluroquinolone resistant (FQR) which likely contributed to the spread of the group strain. However, there is a paucity of data comparing historic clinical isolates prior to the spread of the epidemic strain.

**Table 1.**
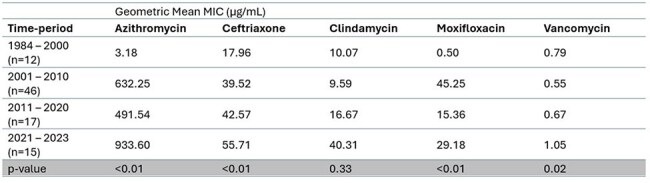
Minimum Inhibitory Concentration for 10 antibiotics for 91 isolates identified at PCR-RT 027 MIC: Minimum Inhibitory Concentration; RT: Ribotype

**Figure 1.**
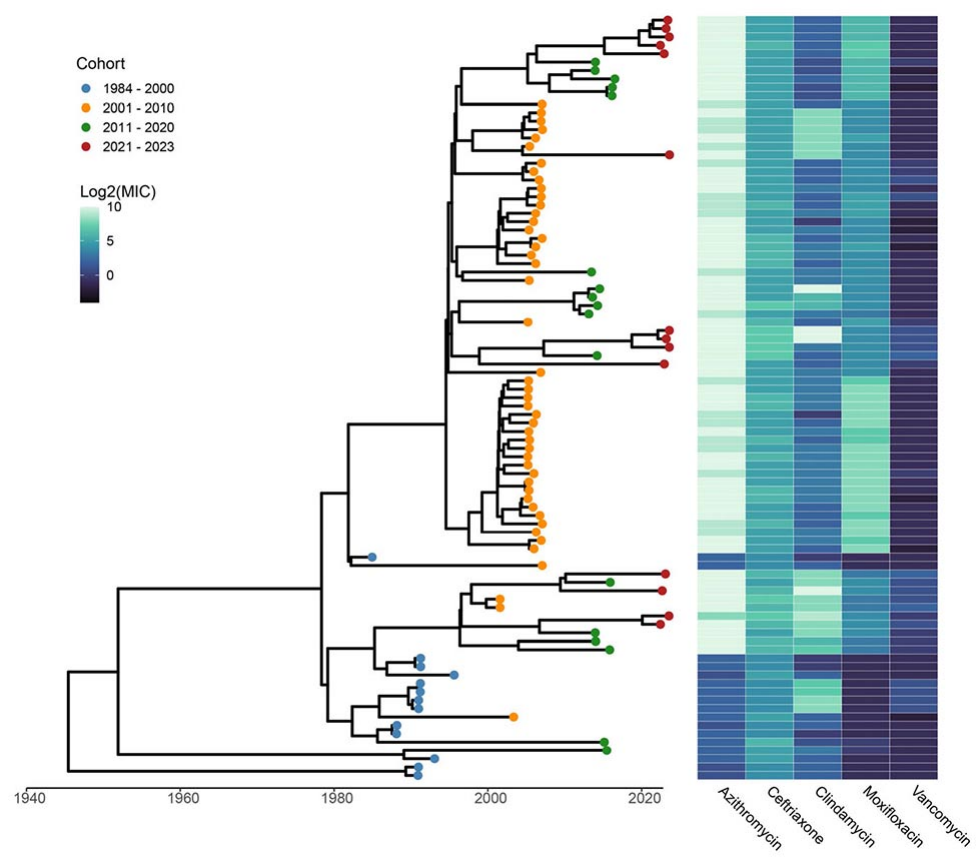
Bayesian time scale phylogenetic analysis with minimum inhibitory concentration changes

**Methods:**

We determined the antimicrobial minimum inhibitory concentration (MIC) for azithromycin (AZM), ceftriaxone (CRO), clindamycin (CLI), moxifloxacin (MXF), and vancomycin (VAN) in 91 clinical isolates previously identified as RT027 collected from 1984 – 2023 from unique patients. Whole genome sequencing was conducted on all 91 isolates to assess for mutations associated with antimicrobial resistance and complete a Bayesian time scale phylogenetic analysis.

**Figure 1.**
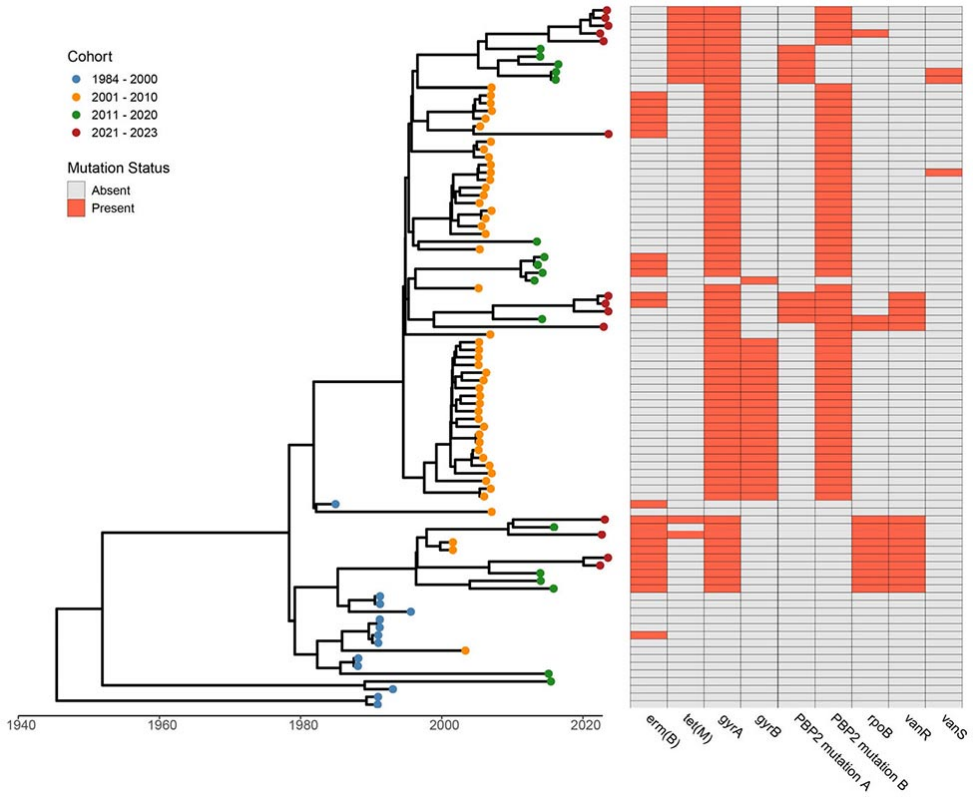
Bayesian time scale phylogenetic analysis with key antimicrobial gene mutations

**Results:**

There was a significant uptrend in the MIC for AZM, CRO, MXF, and VAN over the 40-year period. Notably, the MIC for AZM increased from 3.18 µg/mL to 933.60 µg/mL, the MIC for CRO increased from 17.96 µg/mL to 55.71 µg/mL, and the MXF MIC increased from 0.50 µg/mL peaking from 2001-2009 at 45.25 µg/mL (p< 0.01, for all comparisons). [Table 1] The *gyrA/B* mutations associated with FQ resistance were not present in the historical cohort. From 2001 – 2020, the *gyrA/B* mutation were commonly found with a valine-to-leucine mutation at position 497 (Figure 1/2). Notably, the FQS isolates had only a modest association with FQR isolates from 2001 - 2020, but they do appear to be associated with FQR isolates from 2021 – 2023. These more contemporary FQR isolates more commonly had an *rpoB* mutation which could confer resistance to fidaxomicin

**Conclusion:**

Despite the prevalence of RT027 decreasing over the past 25 years, it still harbors significant mutations which confer antimicrobial resistance. While accrual of mutations can lead to a significant fitness cost to bacteria, continued surveillance is required to ensure that new outbreaks associated with RT027 do not occur.

**Disclosures:**

Andrew M. Skinner, MD, Recursion Pharmaceuticals: Advisor/Consultant

